# Person-centered, non-pharmacological intervention in reducing psychotropic medications use among residents with dementia in Australian rural aged care homes

**DOI:** 10.1186/s12888-020-03033-w

**Published:** 2021-01-13

**Authors:** Daya Ram Parajuli, Abraham Kuot, Mohammad Hamiduzzaman, Justin Gladman, Vivian Isaac

**Affiliations:** College of Medicine and Public Health, Flinders Rural Health South Australia, Flinders University, Po Box 852, Ral Ral Avenue, Renmark, Australia

**Keywords:** Dementia, person-centered care, non-pharmacological intervention, psychotropic medicines, rural Australia

## Abstract

**Background:**

High rates of psychotropic medications are prescribed in aged care homes despite their limited effectiveness and associated adverse effects. We aim to evaluate the changes in prescription patterns for elderly residents with dementia in the ‘Harmony in the Bush Dementia Study’. Harmony in the Bush is a person-centered model of dementia care in nursing homes, based on the principles of Progressively Lowered Stress Threshold and person-centered music intervention.

**Methods:**

Our larger study (12 weeks period) was a quasi-experimental design conducted in five rural nursing homes in Australia. Medication charts (*n* = 31) were collected retrospectively from three rural aged care facilities. Medication data for each resident was collected from a three-month medication charts, pre-intervention, and post-intervention. Fifty-three staff participated in 31 semi-structured interviews and 8 focus groups at post-intervention, and at 1-month and 3-months follow up.

**Results:**

The median age of the participants was 83 years, and 68% of them were female. Polypharmacy was measured in 87% (*n* = 27) of the participants. Hypertension, hyperlipidemia, diabetes, and the Alzheimer’s disease were the major comorbidities identified in residents. None of the residents received more than the maximum dose of psychotropic medications recommended by the guidelines. There was a reduction of 22.4% (77.4% vs 55%) in the use of at least any psychotropic medications, 19.6% (39% vs, 19.4%) reduction in antipsychotics and benzodiazepines (39% vs 19.4%), and 6.5% (42% vs 35.5%) reduction in antidepressants prescription medicines, when comparing residents’ medication charts data covering 3-months pre- and post-intervention, however, these changes were not statistically significant. Additionally, there was a decreasing trend in the use of inappropriate medications. Psychotropic medications were prescribed in up to 43% and anti-dementia medications in 44% of participants for more than 6 months. Three themes extracted from qualitative data include decrease behavioral and psychiatric symptoms of dementia due to medication weaning or dose tapering, other strategies to reduce medication use, and environmental or noise control.

**Conclusions:**

Our findings indicate that the Harmony in the Bush model as a non-pharmacological approach reduces the prescription of psychotropic medications in rural nursing homes as supported by findings from both quantitative and qualitative data.

**Trial registration:**

ANZCTR, ACTRN12618000263291. Registered on 20th February 2018.

**Supplementary Information:**

The online version contains supplementary material available at 10.1186/s12888-020-03033-w.

## The known

High rates of psychotropic medications are prescribed in aged care homes despite their limited effectiveness and associated adverse effects.

**What is new?**

- Higher prevalence of psychotropic medicines dispensing to resident with dementia living in rural nursing homes.

- Non-pharmacological intervention has the potential to reduce the use of psychotropic medicines prescription 3-months pre- and post-intervention.

**Implications**

Harmony in the Bush model as a non-pharmacological approach can reduce the prescription of psychotropic medications and inappropriate medications use in residents living with dementia in rural nursing homes supported by quantitative and qualitative findings.

## Background

An estimated 46.8 million people were living with dementia worldwide in 2015, and it is projected to increase to 74.7 million by 2030 [1]. Dementia is the fifth leading cause of death globally [2] and second leading cause of death in Australia [3]. People with dementia (PwD) present behavioral and psychological symptoms, which are characterized by several behaviors such as aggression, screaming, wandering, restlessness, and symptoms such as anxiety, hallucinations, and depression [4]. These behavioral and psychological symptoms of Dementia (BPSD) are also known as neuropsychiatric symptoms, identified in around 90% of those with dementia, and associated with unmet physical and/or psychological needs as well as the physiological impairment in brain function [4]. Dementia not only decrease the quality of life of PwD [5], but the management of BPSD in dementia builds substantial physical, psychological, social, and financial burden to care providers, families and more broadly, the whole society as reported in previous studies [6, 7].

Psychotropic medicines (antipsychotics, antidepressants, and benzodiazepines) are commonly prescribed for the management of BPSD in PwD despite their limited efficacy and severe adverse effects [8, 9]. Currently, there is a high prevalence of dispensing of these medicines [10, 11]. Surprisingly, only 10% of psychotropic medications prescribed to treat BPSD in PwD has been estimated to be appropriate [12], but these medications are often overprescribed than what has been recommended to be optimal [13]. Antipsychotics have been widely used for long term (≥6 months) with limited monitoring of their adverse effects [14]. Thus, continuous monitoring for effectiveness and potential adverse effects with the aim of discontinuation should always be a central focus while using psychotropic medicines in PwD [15].

Due to fewer risks and adverse effects, Non-pharmacological interventions (NPI) are recommended as a first-line approach to manage BPSD in PwD [16, 17]. Despite a growing body of literature about the effectiveness of NPI approach in the management of dementia [18, 19], there is a lack of consensus in current guidelines for the management of BPSD in PwD [20]. Furthermore, NPI suffered from several challenges, particularly in implementation due to limited efficacy, availability of trained staffs and need of patient-centered approach [21]. In Australia, approximately more than 50% of the residents living in residential aged care facilities (RACFs) have a diagnosis of dementia [22]. The burden of challenges is more abundant for PwD and care givers in rural regions [23], where it is more difficult to attract and retain a skilled workforce and there is a lack of training resources compared to metropolitan areas. Additionally, shortage of skilled human resources and dementia-specific services has been reported in rural areas [24].

According to the most updated report of the Royal Commission in 2019, depending on patients’ profile, psychotropics medicines would be appropriate and effective if used in lowest doses and as per the guidelines, but there is a growing concern about their use in aged care homes in Australia and overseas [25]. Our larger study, the Harmony in the Bush, used a quasi-experimental design, to evaluate the impact of a multi-factorial NPI in five Australian rural aged care homes. The Harmony in the Bush is a co-designed dementia care model that integrates person-centered care plan based on the principles of Progressively Lowered Stress Threshold (PLST), and person-centered music intervention to reduce BPSD in resident with dementia (RwD) as well as reduction in staff stress in caregiving [26, 27]. This sub-study aims to evaluate the changes in prescription patterns of psychotropic medications in RwD, who participated in our larger study.

## Methods

This study is presented following the Strengthening of Reporting of Observational Studies in Epidemiology (STROBE) guidelines [28]. The term psychotropic medications represent the combination of antipsychotic, antidepressants, and benzodiazepines group of medications in our study. Polypharmacy status was defined if ≥5 medications were taken daily by RwD [29] in our study.

### Study design and settings

The larger study ‘Harmony in the Bush’ (2017–2019) was a quasi-experimental design (nonrandomized, pre-post intervention) without a control group. It was conducted in five Australian rural aged care homes (three in Queensland and two in South Australia). Among the five nursing homes, two were privately owned, one was public funded, one Aboriginal, and one not-for-profit organization. The larger study encompassed a 12-weeks period (1-week baseline assessment, 3-weeks PLST and person-centered dementia care training to the participating staff, and 8-weeks person-centered music intervention along with person-centered care based on the PLST principles) comparing pre-and-post intervention. Medication charts (*n* = 31) were collected retrospectively from three Australian rural aged care facilities through contacting the facility managers.

### Participants

Participants included eligible RwD and aged care staff (clinical managers, directors of nursing, registered nurses, enrolled nurses, life-style managers, and care workers) from the participating nursing homes. The participating residents had a diagnosis of dementia within the Diagnostic and Statistical Manual of Mental Health Disorders 5 (DSM-5) [30].

### Intervention: staff training and person-centered music

Detail description of the intervention program is described in another publication from our larger study [26]. Staff training (approximately 2 h for each staff up to 3 weeks) was provided by a nursing educator/investigator about personalized dementia care based on the PLST principles. Person-centered dementia care mainly focusses on the maintenance of individual daily routines, organize group activity to minimize stress stimuli, afternoon nap, and residents were encouraged to implement their sleep and wake cycles to avoid fatigue. Activities were designed considering the previous history and current cognitive and functional abilities of the residents. To promote these activities, two staff in each facility were appointed and trained as change champions.

A list of preferred songs/composition which best suits for the residents were selected in consultation with staff members, families, and professional musicians. The person-centered music intervention is of 20–30 min; two sessions per day in the morning and afternoon over 8 weeks. The quality of music and volume adjustment was tested for headphones and speaker used before person-centered music intervention. Group music activity was added to the intervention at 4 weeks conducted for 45–50 min; 2 h a week over a duration of 4 weeks. Musicians pay attention to culturally appropriate songs for each resident before running group music activity.

### Data collection

Medication charts (*n* = 31) were collected retrospectively for each resident covering 3-months pre- and post-intervention from the three Australian rural nursing homes. Medication details (name, doses, frequency, and duration of use), demographic characteristics and comorbidities of the residents were recorded. Opinions of staff about the impact of the intervention on medication use were received from 53 staff who participated in 31 one-to-one semi-structured interviews and 8 focus groups conducted at post-intervention, and at 1-month and 3-months follow up. Questionnaires used for qualitative interview are illustrated in supplementary material.

### Statistical analysis

Statistical analysis was performed using IBM SPSS Statistics for Windows (Version 25.0.0.1. Armonk, NY: IBM Corp) for quantitative data. Results are presented as frequency and percentages for categorical variables whereas median (IQR) for continuous variables. The normal distribution of the numeric variables was confirmed using Shapiro–Wilk test (*P* > 0.05). Differences in medication use at pre- and post-test was evaluated using Mann–Whitney U test. Probability values of *p* < 0.05 were chosen to indicate a statistically significant difference. For the analysis of the qualitative data of in-depth interviews, NVivo 12 software was used. Two researchers (DRP and AK) imported the data into the software independently. Thematic analysis technique described by Braun and Clarke (2006 and 2014) was utilized in coding and analyzing the data [31, 32]. We followed several steps of thematic analysis: (i) listen the audios and read the transcripts for data familiarization; (ii) open coding that provided an insight for each theme related to medication reduction; (iii) topic coding to generate derivative categories (i.e. nodes) from each transcript; (iv) categorization of codes into nodes or candidate sub-themes; (v) candidate sub-themes and themes were discussed and reviewed; and (vi) define sub-themes and themes. Extracted themes by each researcher were discussed and critically analyzed in the weekly meetings of the Harmony in the Bush project.

## Results

### Characteristics of the participants

An overview of the different psychotropic medications, defined according to the Australian Medicines Handbook, prescribed to the participants of our study is illustrated in Table 1. The doses of different psychotropic medications prescribed to participants did not exceed the maximum dose recommended in the Australian Medicines Handbook. Medication charts of 31 patients were reviewed. The median age of the participants was 83 years, and 68% were female. Polypharmacy was measured in 87% of the participants, and the median number of medications use at baseline was 8 (Table 2). Majority of participants (35.5%) do not have formal education followed by secondary schooling (32.3%) and primary schooling (23%). Hypertension, hyperlipidemia, diabetes, eye disease and Alzheimer’s disease were the major comorbidities identified in RwD (Table 2).

**Table 1:** Different psychotropic medications prescribed to the participants of the Harmony in the Bush study defined by the Australian Medicines Handbook

Antipsychotics	Antidepressants	Benzodiazepines
Risperidone (0.25 mg OD and BD, 0.5 mg OD and BD, 1 mg OD) Olanzapine (2.5 mg OD, 5–10 mg TDS, 5 mg OD) Quetiapine (12.5 mg BD and TDS, 25 mg OD, BD and TDS, 50 mg OD, 75 mg OD, 100 mg OD)	*SSRIs* Escitalopram (5 mg OD, 10 mg OD) Sertraline (100 mg OD) *Tricyclic antidepressants:* Amitriptyline (50 mg OD) *Serotonin and noradrenaline reuptake inhibitors:* Venlafaxine (150 mg OD) *Other antidepressants:* Mirtazapine (15 mg OD, 30 mg OD	Oxazepam (5 mg BD, 7.5 mg OD, BD and TDS, 15 mg TDS) Temazepam (10 mg OD) Clonazepam (0.5 mg OD) Diazepam (2.5 mg OD)

*OD* Once daily, *BD* Twice a day, *TDS* Three times a day, *SSRIs* Selective serotonin reuptake inhibitors

Accessed date; 21,022,020; https://amhonline.amh.net.au/chapters/psychotropic-drugs?menu=banner

**Table 2:** Baseline demographic characteristics of residents with dementia

Characteristics	N (%)
Gender	
Male	9 (29.0)
Female	21 (68.0)
Age in years, median (IQR)	83 (78–89)
Average duration of stay in aged care centers (years), median (IQR)	2 (1–4)
Polypharmacy	27 (87.1)
Number of medications used, median (IQR)	8 (5–11)
Born in Australia	19 (61.3)
Born oversea	7 (23.0)
Highest level of qualification	
No formal education	11 (35.5)
Primary schooling	7 (22.6)
Secondary Schooling	10 (32.3)
University Graduate	1 (3.2)
Comorbidities	
Hypertension	19 (61.3)
hyperlipidemia	13 (42.0)
Osteoarthritis	12 (39.0)
Diabetes	10 (32.3)
GORD	10 (32.3)
Eye disease	9 (29.0)
Alzheimer’s disease	6 (19.4)
Thyroid disease	6 (19.4)
Osteoporosis	6 (19.4)
Asthma	5 (16.1)
CRF	5 (16.1)
COPD	5 (16.1)
Atrial fibrillation	4 (13.0)

*IQR* Interquartile range, *GORD* Gastroesophageal reflux disease, *CRF* Chronic renal failure, *COPD* Chronic obstructive reflux disease

### Psychotropic medications use among the participants

There was a reduction of 22.4% (77.4% vs 55%)) in the use of at least any psychotropic medications 19.6% reduction in antipsychotics (39% vs 19.4%) and benzodiazepines (39.0% vs 19.4%) and 6.5% reduction in antidepressants prescriptions (42% vs 35.5%) when comparing residents’ medication charts data covering 3-months pre- and post-intervention, however, was not statistically significant (Table 3). The prescription of anti-dementia medications remained unchanged at 26% from pre- to post-intervention. For the individual class of antipsychotics, risperidone, olanzapine, and quetiapine were the major medications prescribed. Similarly, mirtazapine and escitalopram were prescribed in the antidepressants group, oxazepam and temazepam in the benzodiazepines group and memantine and donepezil as antidementia medications.

**Table 3:** Comparison of utilization of medications use between baseline and post intervention

Use of Medications (%)	Pre-intervention (***n*** = 31)	Post intervention (***n*** = 31)	***P***-value
At least any psychotropics	24 (77.4)	17 (55.0)	0.106
Antipsychotics	12 (39.0)	6 (19.4)	0.161
Risperidone	5 (16.1)	4 (13.0)	1.0
Olanzapine	4 (13.0)	1 (3.2)	0.354
Quetiapine	4 (13.0)	4 (13.0)	1.0
Antidepressants	13 (42.0)	11 (35.5)	0.795
Mirtazapine	3 (8.0)	3 (8.0)	1.0
Escitalopram	6 (19.4)	5 (16.1)	1.0
Benzodiazepines	12 (39.0)	6 (19.4)	0.161
Oxazepam	6 (19.4)	3 (8.0)	0.473
Temazepam	3 (8.0)	3 (8.0)	1.0
Anti-dementia medications	8 (26.0)	8 (26.0)	1.0
Memantine	2 (6.5)	2 (6.5)	1.0
Donepezil	6 (19.4)	6 (19.4)	1.0
Combination of antipsychotics and antidepressants	6 (19.4)	4 (13.0)	0.731
Combination of antipsychotics and benzodiazepines	5 (16.1)	1 (3.2)	0.195
Combination of antipsychotics and anti-dementia	5 (16.1)	4 (13.0)	1.0
Combination of antipsychotics and donepezil	3 (8.0)	3 (8.0)	1.0
Combination of antipsychotics and memantine	2 (6.5)	2 (6.5)	1.0

There was a decreasing trend in the concomitant use of antipsychotics and benzodiazepines prescription, antipsychotics and antidepressants, and antipsychotics and anti-dementia medications (Table 3). Overall, there was no statistically significant difference in reduction patterns. Most of the residents were prescribed psychotropics and anti-dementia medications for > 6 months duration (Table 4). Psychotropic medications were prescribed in up to 43% and anti-dementia medications in 44% of participants for more than 6 months.

**Table 4:** Duration of use of psychotropic medications in patients with dementia

Medication class	< 3 months	3–6 months	> 6 months
Antipsychotics (*n* = 17)	7 (41.17)	3 (17.64)	7 (41.17)
Antidepressants (*n* = 14)	3 (21.42)	5 (35.71)	6 (42.85)
Benzodiazepines (*n* = 12)	5 (41.66)	2 (16.66)	5 (41.66)
Anti-dementia (*n* = 9)	2 (22.22)	3 (33.33)	4 (44.44)

### Major themes from in-depth interviews with aged care staff

Three themes emerged from the qualitative data analysis include decrease behavioral and psychiatric symptoms of dementia due to medication weaning or dose tapering, other strategies to medication use, and environmental or noise control.

#### Less behavioral and psychiatric symptoms due to medication weaning or dose tapering

Participants mentioned that doctors embedded medication weaning as a strategy of using less medications. One participating carer explained that Risperidone and Oxazepam were off due to less BPSD symptoms. Psychotropic medications can make residents drowsy, sleepy, shaky, and agitated. Dose tapering or cessation of psychotropic medications have several benefits to the residents if clinically justified as their use is associated with several adverse effects.

*Yeah, I would need to look at that, yeah. We don’t use a lot of antipsychotic medication anyway. Um, we have a little bit – a little bit regular for you know, some residents to manage their mental health illnesses. But we don’t use a lot of PRM antipsychotic, and I do – I do measure that regularly from time to time just to measure where we’re at with antipsychotic medication … .* [ Staff 1]

*Yeah, there’s lots of medication weaning, doctors are reducing the dose of medication. So, that’s really happening, good for these people, yeah, is working on that a lot … .* [Staff 2]

*Well, medications constantly change. Um, there have been a couple that we’ve been able to take off of things like risperidone and oxazepam which is good. Um, so that’s always a plus when we can do that … because their behaviors aren’t there anymore, they are not needing … it, so, ah, but it’s, yeah, that’s a few of them. Like I said, other than that, their medications change, but, um … . only with that risperidone and oxaz, I’ve seen a reduction in that … but … but nothing* … . [Staff 3]

*Um, well, we do medications, um, I don’t really have a lot of things to say about medications because, um, that’s – as a for now that’s out of my scope. But yeah, ah, at times I have … In general, yeah, I would say it – it – it effects, you know, them and their behaviors as well. Um, even with regards to like falls and stuff because, you know, some medications make them really drowsy. So, it’s yeah, like some of them I could see like – because they really that sleepier and shaky. So, um, yeah, or some of them, I guess it’s just due to medications as well that doesn’t suit, yeah, you know, them, and they became more agitated. So that’s just* … … . [Staff 4]

#### Other strategy to reduce medication use

Clinical Managers mention that residents have been using less antipsychotics and there is a measure to check it regularly. Carers were unaware of how much medications the residents have been using. Indeed, it is good to have their opinion about potential less use of medications due to less behavioural and psychiatric symptoms. Two Residents were taken off medications like Risperidone. Use of sleeping tablets (benzodiazepines) was also reduced. Residents were offered a drink and Weet-Bix instead of the sleeping tablet as an alternative to medications. As a result of less use of sleeping tablets, residents exhibited less symptoms of being sleepy and hung over, and there was a reduction of falls. Residents receive pain relief and behavioral management medications like Oxazepam. A Clinical Nurse Manager has indicated that she has noticed a marked reduction in the use of medications after the intervention was implemented. Residents showed less behavioural and psychiatric symptoms of dementia with the opportunity of a resting period and music as a non-pharmacological intervention. Ultimately, because of such less behaviors, residents were given less medications like Oxazepam.

*I — I’m not a nurse. I do help with medication, so I don’t see all the medications. Um, again, I think some of our extreme cases, we try to use other strategies before the medication. Some people — it — unfortunately, it seems as if every strategy you use, enact, it doesn’t work on everybody. So - - - medication may be the last resort. Um, but it’s those extreme cases that we can’t always cut through from what I’ve seen. Those are the ones that we use medication with, and I don’t know for sure, but I don’t think it’s changed the amount as such. Um, and again, I don’t know, but - Uh, I can’t give you an accurate answer because I’m not here - - - like, I’m not with the nurse all the time - - - I mean I would expect less medication used because of a lower, uh, lower stressors and lower reactive behaviors. But again, I know, through my experience with the people that are more extreme cases, they still get worked up. They still get worked up. They still, um, at times, need medication. Some of them more moderate or lower end of the dementia, I would think that we could probably stick with more of the strategies that aren’t medication based. I mean I — I don’t know for sure but that’s just, from my, uh, my experience, yeah, I would — I would like to think that would be less. But I don’t really know. I can’t give you an exact number. … …* [Staff 5]

*Um, we've actually reduced, I think, two people are now off Risperidone all together, that we’ve just recently stopped that we were tapering it down and we’ve just, recently they’ve ceased it. We still do have a couple that need it, because they're still in that stage, but we haven’t worked with them quite as intensively just yet … . Yep. Um, there aren’t too many that have sleeping tablets overnight anymore, we used to get people, and they would automatically just give them a sleeping tablet if they were up. Now they give them a drink and Weet-Bix and they don’t need the sleeping tablet, which is really good. Because then you haven’t got people in the morning who are drugged out and sleepy and hung over and falling … ...* [Staff 6]

**…** *um, obviously we have – we have I guess given less pain relief, given less behavior management medication like Oxazepam and all that. So there has been some because I keep an eye on progress notes while in care. So, I have seen that there was a lot before, but it has decreased. We used to give medications a lot to some people, and now obviously introducing a rest period and the music therapy combined, I think it had an impact on the way they behaved. As I said, Oxazepam we used to give for the behavior management. … .* [Staff 7]

#### Environmental or noise control

The fewer behavioural and psychiatric symptoms of the residents were well reflected by the quiet environment, as noticed by the Lifestyle Manager. There was an anticipation that the use of medications would have been less due to the quiet environment. Changes in behaviors of the RwD can be very confronting and unpredictable, but a carer can notice those change in behaviors of the residents.

*I don’t know whether there has been any. I would love to have seen medications have gone down through the floor, because of the quiet, very quiet environment. I would love to have seen that. I don’t know whether there has been … . I don’t know whether there has been any. I would love to have seen medications have gone down through the floor, because of the quiet, very quiet environment. I would love to have seen that. I don’t know whether there has been. Because I’ve come – I’ve just been back and watched, and I’ve been seeing ice-creams being handed out to people. So, I don’t know, but I would love to know whether the medications had dropped during that period. And then it may be worthwhile … ...* [Staff 8]

In summary, there was a reduction in the use of psychotropic medications as described by participating staff. In some situations, the dose was lowered and some medications, for example risperidone (antipsychotic) and oxazepam (benzodiazepine) were not given because of less behavioural and psychiatric symptoms exhibited by the residents. As the residents were offered a rest period and time to listen to their preferred music, there was less need to use medications.

## Discussion

This study evaluated the changes in prescription patterns of psychotropic medications used in RwD from Australian rural nursing homes from the larger study, ‘Harmony in the Bush: a person-centered dementia care model’. There was a reduction trend in the use of psychotropic medications and use of inappropriate medications when comparing residents’ medication charts data covering 3-months pre- and post-intervention. None of the study participants were prescribed more than the maximum dose of psychotropic medications recommended by Australian Medicine Handbook which we confirmed by checking the maximum dose prescribed for each medication. Nearly 50% of the residents were receiving psychotropic and anti-dementia medication for more than 6 months. Qualitative findings clearly complement those observed from the quantitative analysis, for the less use of psychotropic medications in RwD; thus, a confirmation of the effectiveness of the Harmony in the Bush dementia care model.

In our study, the prevalence of antipsychotics use was 39% at pre-intervention whereas a literature review conducted by Westaway et al. 2018 in Australia reported a prevalence ranging from 13 to 42% [33]. A previous study in 150 aged care homes (*n* = 12,157) in seven Australian states and territories reported the prevalence of antipsychotics use as 22%, and benzodiazepines 22% [34]. A more recent study in Australia (*n* = 322,120) found that 50% of the participants had a diagnosis of dementia, and the prevalence of antipsychotic dispensing was 21.3%, benzodiazepines 30.5% and antidepressants 38% [35]. Comparing to above two larger studies [11, 34], the prevalence of psychotropic medicines prescription was higher in our study. Unique aspect of our study is that it was conducted in rural settings. The residents with a diagnosis of dementia in Australia are exposed to polypharmacy, with an average exposure of nine to ten regular medications (44). A similar pattern of polypharmacy was observed in our study, a potential risk for quality use of medicines.

The NPI to manage BPSD in PwD have several benefits such as limited harmful effects compared to medical therapy, highly acceptable, feasible, and designed for person-centered model of care [36]. A systematic review of randomized controlled trials (RCTs) demonstrated that most successful NPI is a music therapy, but the quality of evidence is quite low [37]. Another more recent systematic review of RCTs showed that NPI were not effective to reduce BPSD symptoms except improvement in activities of daily living in patients with moderate to severe dementia [38]. Such a heterogenicity about the effectiveness of NPI reflects a research gap in this field. Meanwhile, our study mainly focused on NPI approach including the PLST-based patient-centered care plans development and intervention, creation of the low-stress facility environment and patient-centered music intervention. Environmental factors, for example, location, light, sound, and the surrounding atmosphere, play a significant role in the development of wandering in PwD [39]. Noise control to create a quiet environment may reduce anxiety, and agitation behavior in PwD [40]. After the PLST training participating staff pay attention to reduce noise coming from television, alarms, bells or telephones, mealtime noise, bathroom noise, light adjustment during sleep and external noise, for example, mowing during rest time to create a good acoustic environment for residents. The opportunity to be involved in person-centered music provided meaningful social engagement. RwD in the current study showed less BPSD and ultimately a less exposure to psychotropic medications.

Several studies attempted to implement strategies for management of BPSD and reduction in the use of psychotropic medications use in RACFs, but these efforts have not always been successful. For example, a RCT was successful in significant reduction (19%) of antipsychotics use in RwD living in aged care homes at 12 months, but there was no significant reduction of agitated or disruptive behaviors [41]. The intervention utilized consisted of providing training to staff about patient-centered approach, behavioral management techniques and reminiscence therapy. Another more recent study, RedUSE program, achieved significant reductions in the proportions of prescription of antipsychotics by 13% and benzodiazepines by 21% [34]. The RedUSe study implemented a multi-component intervention consisted of psychotropic medication audit and feedback, staff education, and interdisciplinary case review, but this study did not explain how many patients recruited have had dementia and the impact of the intervention on management of BPSD. Our study showed quite similar pattern of reduced use of psychotropic medicines as well as reduction of BPSD symptoms [26].

Psychotropic medications use in PwD need regular review and assessment for potential tapering and cessation [42]. However in real life, clinicians are often reluctant to implement a reduction or discontinuation plan due to limited staffing and other resources [43]. Our qualitative findings showed that residents were offered a drink and Weet-Bix instead of the benzodiazepines tablets for sleeping. As a result of less use of sleeping tablets, residents have beneficial effects, including not waking up sleepy in the morning, and/or experiencing hungover and falling episodes. Doctors embedded medication weaning as a strategy of using fewer medications, and two residents were taken off Risperidone use. Dose tapering or cessation of psychotropic medications had several benefits if clinically justified. Residents become more drowsy, sleepy, shaky, and agitated due to use of these medications. Less BPSD symptoms were exhibited by the residents due to the opportunity for afternoon sleep and music as an alternative non-pharmacological approach to medication use. It is highly likely that resident may feel less fatigue due to afternoon sleep favored by noise control. All this evidence from our qualitative data is consistent with the reduced use of psychotropic medications from quantitative findings.

Potentially inappropriate medications are a group of drugs that their potential harms overweight their clinical benefits [44]. Concomitant use of antipsychotics and antidepressants is considered to be of inappropriate practice, was prescribed to 15.7% of participants in an earlier study [45], while it was 19.4% at pre-intervention in the current study. Interestingly, there were less RwD receiving the combination use of different psychotropic and anti-dementia medications in the current study when comparing residents’ medication charts data covering 3-months pre- and post-intervention A total of 65% of participants were using antipsychotics for > 3 months in an earlier study [46]. Furthermore, in the same study, the duration of use exceeded more than 12-week for most of the residents. Prolonged use of benzodiazepines along with antidepressants has also been reported previously in 40% of the study participants [47]. Our findings showed evidence of long term use of psychotropic and anti-dementia medications for ≥6 months, although current guidelines stipulate review and withdrawal within 12 weeks [48].

Our study was conducted in rural nursing homes which are experiencing substantial challenges in recruitment and retention of skilled staff as additionally, there are limited resources available for professional development compared to metropolitan areas [49]. In a previous study, nursing staff working in rural settings demonstrated that that NPI management of BPSD in PwD do not fall under their responsibility [50]. Our larger study had identified that an effective model of care to RwD in rural settings should include the voice of family members and residents while designing care plans, providing sufficient training to staff, and a supportive leadership and organizational culture for work-life balance of the staff [27]. Particularly important, our study reinforces that positive outcomes can be achieved by providing dementia-focused training to the existing staff.

Despite of a reduction trend in the use of psychotropic medicines by implementation of a patient centered NPI approach, our results were not statistically significant. Nevertheless, in clinical practice, a non-significant outcome does not always mean the treatment was not clinically effective as small sample sizes had a substantial impact [48]. Additionally, another potential reason to support an association of small sample size for the non-significant reduction in the use of psychotropic mediations is that all the participating staff in semi-structured interviews and focus group highlighted a less need for medications due to reduction in BPSD of the residents. Our findings suggest that Harmony in the Bush model was a feasible NPI for dementia care in RwD living in rural nursing homes and can reduce the prescription of psychotropic medications and inappropriate medications use from pre- to post-intervention; as supported by findings from both quantitative and qualitative approaches.

Based on a report of a systematic review, currently available intervention to reduce the use of antipsychotics in PwD in aged care homes are effective only for short term [51]. Future studies should focus on large scale RCTs in PwD living in aged care homes to evaluate the impact of staff training and implementation of medication review [52], which is likely to have significant contribution on reduction of BPSD and promote the quality use of psychotropic medicines.

### Strength and limitations

The major strength of our study is that it was conducted in rural aged care homes. Reduction in use of medications has been demonstrated in a mixed method study design. We analyzed medication chart data, and therefore had no information about prescriptions issued and dispensed, but not used. The major limitation of our study is that we were unable to obtain medication charts from the two other sites where our larger study was conducted. The interpretation of the findings of the current study are limited by small sample size.

## Conclusions

Presence of polypharmacy, comorbidities, and high dispensing of psychotropic medications in residents with dementia in rural nursing homes was consistent with previous studies. But more importantly, our study findings indicate that the Harmony in the Bush model as a non-pharmacological approach reduces the prescription of psychotropic medications and inappropriate medications in aged care homes as supported by findings from both quantitative and qualitative data.

## Supplementary Information

**Additional file 1.**

## Data Availability

The datasets used and/or analyzed during the current study are available from the corresponding author on reasonable request.

## Abbreviations

PLST: Progressively Lowered Stress Threshold
PwD: Patient with dementia
BPSD: Behavioral and psychological symptoms of dementia
RwD: Resident with dementia
NPI: Non-pharmacological interventions
RCTs: Randomized controlled trials
