# Correction to: Person-centered, non-pharmacological intervention in reducing psychotropic medications use among residents with dementia in Australian rural aged care homes

**DOI:** 10.1186/s12888-021-03141-1

**Published:** 2021-03-17

**Authors:** Daya Ram Parajuli, Abraham Kuot, Mohammad Hamiduzzaman, Justin Gladman, Vivian Isaac

**Affiliations:** College of Medicine and Public Health, Flinders Rural Health South Australia, Flinders University, Po Box 852, Ral Ral Avenue, Renmark, Australia

**Correction to: BMC Psychiatry 21, 36 (2021)**

**https://doi.org/10.1186/s12888-020-03033-w**

Following the publication of the original article [1], some words were incorrectly used (in two places) and few errors were identified in the reference section.

The changes have been highlighted in **bold typeface**.

Statistical analysis:

The normal distribution of the numeric variables was **checked** using Shapiro–Wilk test (*P* > 0.05).

Discussion:

The residents with a diagnosis of dementia in Australia are exposed to polypharmacy, with an average exposure of **nine** regular medications **(35)**.

Concomitant use of antipsychotics and antidepressants is considered to be of inappropriate practice, was prescribed to 15.7% of participants in an earlier study **[35]**, while it was 19.4% at pre-intervention in the current study.

A total of 65% of participants were using antipsychotics for > 3 months in an earlier study **[45]**.

Prolonged use of benzodiazepines along with antidepressants has also been reported previously in 40% of the study participants **[46]**. Our findings showed evidence of long term use of psychotropic and anti-dementia medications for ≥6 months, although current guidelines stipulate review and withdrawal within 12 weeks **[47]**. Our study was conducted in rural nursing homes which are experiencing substantial challenges in recruitment and retention of skilled staff as additionally, there are limited resources available for professional development compared to metropolitan areas **[48]**. In a previous study, nursing staff working in rural settings demonstrated that that NPI management of BPSD in PwD do not fall under their responsibility **[49]**.

Nevertheless, in clinical practice, a non-significant outcome does not always mean the treatment was not clinically effective as small sample sizes had a substantial impact **[50]**.

The author group has been updated above and the original article [1] has been corrected.
