# Supplementary material for: Person-centered, non-pharmacological intervention in reducing psychotropic medications use among residents with dementia in Australian rural aged care homes
Source: BMC Psychiatry. 2021 Jan 13;21:36. doi: 10.1186/s12888-020-03033-w (PMC7805083; doi:10.1186/s12888-020-03033-w)
Supplement: Supplementary file 1 — Additional file 1. [file 12888_2020_3033_MOESM1_ESM.docx]

**Supplementary file**

**Care Staff Focus Group Discussion (FGD):** Questions and discussion points

These questions and discussion points are provided as a guide for the FGD. The discussion will not be limited to these questions. The focus groups will be audiotaped with participant consent and transcribed for analysis and reporting purposes

Think back over the period you have participated in the program and tell us what was most useful

o to the resident

o to you

o to the residential home

Give me a picture of how the program influenced the elderly persons overall health

Tell me more about their

• physical health and functioning – Give me an example

• agitation

• sleep

• memory and cognition – Give me an example

• mood – Give me an example

• engagement with family and friends – Give me an example

What is the impact of changes in care-plans using PLST approaches?

What is the impact of personalised music/arts?

Would you like to continue to engage in PLST and arts programmes? If so how?

What changes/additions would make the program better?

•any other comments?

**Interview questions for Aged Care Staff**

These questions are provided as a guide for the Interviews. The discussion will not be limited to these questions. The interview will be audiotaped with participant consent and transcribed for analysis and reporting purposes

Welcome – Introduction and consent

• Can you tell me about your participation in the ‘harmony in the bush’ research study?

• What is your impression on the educational activities you have participated?

• Have you encountered difficulty during the study? Please explain.

• What changes have you noticed in the way staff provide care for the residents involved in the study?

• What aspects of the resident’s well-being have the program significantly contributed? o Probes: Physical, Behaviour, Sleep, Medications, Social wellbeing and Living environment

• What is the impact of the program on job stress, job satisfaction and staff wellbeing?

• What aspects of the care you currently provide could be improved?

• Suppose that you were in charge and could make one change that would make the services better. What would you do?

• Of all the things we've talked about, what is most important to you? Did we miss anything you would like to comment about?

I think we’ve come to the end of our questions. Let me say thank you for your honest opinions. We really appreciate your help.
